# Sustainable artificial intelligence in finance: impact of ESG factors

**DOI:** 10.3389/frai.2025.1566197

**Published:** 2025-03-06

**Authors:** Paolo Giudici, Lunshuai Wu

**Affiliations:** ^1^Department of Economics and Management, University of Pavia, Pavia, Italy; ^2^Department of Business, Ningbo University, Ningbo, Zhejiang, China

**Keywords:** explainability, ESG scores, credit ratings, gradient boosting, Shapley values

## Abstract

There is a growing concern about the sustainability of artificial intelligence, in terms of Environmental, Social and Governance (ESG) factors. We contribute to the debate measuring the impact of ESG factors on one of the most relevant applications of AI in finance: credit rating. There is not yet conclusive evidence on whether EGS factors impact on credit rating. In this paper, we propose several machine learning models to measure such impact, and a set of metrics that can improve their ability to do so. In this way, machine learning models and, more generally, decisions based on artificial intelligence, can become more sustainable.

## 1 Introduction

Artificial intelligence applications can bring many opportunities and increase efficiency, but can also increase the risks of harms, to the individuals, the society and the environment. For this reason, there is a growing concern about the sustainability of artificial intelligence applications, from the environmental viewpoint (e.g. in terms of energy consumption) but also in terms of its social impacts (e.g. fairness and discrimination) and in its governance (e.g. controllability and cybersecurity).

In the financial sector, artificial intelligence methods are increasingly applied to determine credit ratings, the fundamental information on which credit lending is based. Although the sustainability of credit ratings suggests that they should depend on Environmental, Social and Governance factors, the empirical results available in the literature do not bring conclusive evidence that this is the case.

The lack of a conclusive evidence depends on the fact that it is not easy to demonstrate the relationship between ESG factors and credit ratings. First of all, it is not clear whether ESG factors do impact credit ratings in the short term. Second, the effect of ESG scores on credit ratings may be non linear, and difficult to capture. Third, ESG scores are not standardized: Dorfleitner et al. (2015) showed little convergence between different ESG ratings and, more recently, Abhayawansa and Tyagi (2021) provided evidence of the low correlation between ESG ratings issued by different providers.

We believe that machine learning models may be useful to capture the relationship between ESG scores and credit ratings, as they can capture nonlinearity. Furthermore, ensemble machine learning models could mitigate the lack of standardization by providing model averaged estimates, as shown in Agosto et al. (2024). A problem with the application of machine learning methods in finance is that, while accurate, they may lack explainability and robustness, as described in the recently proposed SAFE machine learning framework (Babaei et al., 2025). To fill this gap, in this paper we propose ensemble machine learning models that: (i) can model the non-linear relationship between ESG scores and credit ratings; (ii) can average the results from different models; iii) can be made explainable and robust.

The remainder of this paper is arranged as follows: Section 2 contains some theoretical background on ESG factors; Section 3 presents a literature review on the relationship between ESG factors and credit ratings, and details our research hypotheses; Section 4 introduces the proposed approach; Section 5 presents an application of the methodology to a sample of companies asking for credit, Section 6 discussed the obtained results and, finally, Section 7 concludes.

## 2 Background

The stream of literature closest to our work is in the field of Corporate Social Performance (CSP), which aims to evaluating the degree to which companies are sustainable, that is, how they conduct their business activities in relation to external stakeholders while considering economic, environmental, social, and time factors (Lozano, 2012; Lozano et al., 2017; Muñoz-Torres et al., 2019). We follow this approach, and consider Environmental, Social and Governance (ESG) factors as a proxy for the sustainable behavior of companies.

Environmental factors (E) relate to the impact on the environment deriving from the production of goods or services and include carbon emissions, preservation of the natural environment, biodiversity protection, waste and water management (Commission, 2024; Times, 2024; Robeco, 2024). A company that operates with minimal environmental impact may reduce the likelihood of future scandals, legal actions, and losses associated with legal claims and may benefit from a better reputation and lower risks (Fafaliou et al., 2022).

Social factors (S) refer to the impacts of companies on society, including issues of employee satisfaction, diversity, inequality, gender gap, protection of youth and children, investment in human capital and community development, as well as human rights (Commission, 2024; Van Duuren et al., 2016).

Governance factors (G) measure the quality and effectiveness of corporate governance. Shortcomings in governance have historically been the cause of major scandals and crises, such as the Enron crisis in the US, Volkswagen in Germany, Parmalat in Italy, and the banking crisis of 2007–2008 (Shin et al., 2022; Soltani, 2014). Improved governance practices can drive more sustainable and balanced company growth, which in turn supports more sustainable economic development (Adams and Mehran, 2012; Esteban-Sanchez et al., 2017).

The above factors are the basis for investment decisions and drive investors' choices regarding which companies to finance through equity or debt. To improve the interpretability of ESG, specialized companies (including rating agencies) have started to provide measures and proxies for ESG behavior, publishing ESG ratings or ESG scores that convey the level of sustainability of companies and the degree of accountability of these companies on ESG aspects (Scalet and Kelly, 2010; Avetisyan and Ferrary, 2013).

Each rating provider collects information from different sources (company reports, news, stock exchange information, etc.) and applies proprietary methodologies to combine this information and produce a summary measure of ESG behavior. Unfortunately, different methodologies yield different measurements, which often produce divergent results (Dorfleitner et al., 2015; Abhayawansa and Tyagi, 2021; Dimson et al., 2020; Billio et al., 2021) leading to a lack of standardization.

The significance of ESG metrics is expected to increase in the future, with ESG ratings potentially influencing investors' decisions, firms' access to financing for their investments, and their ability to pursue sustainable business models. Consequently, understanding the impact of ESG ratings (whether and how) on creditworthiness represents a critical managerial and policy challenge.

## 3 Literature review and research hypotheses

The topic of sustainable artificial intelligence is recent, especially from a quantitative viewpoint, and few papers are present in the literature. Among them, the following are related to our work.

Nishant et al. (2020) argues that artificial intelligence (AI) not only reshapes business operations and industrial landscapes but also hold the potential to address critical societal issues, including sustainability. In the face of the environmental degradation and the climate crisis, cutting-edge innovative solutions are particularly necessary. To foster novel research and practical applications of AI in environmental protection, AI could assist in developing organizational processes and personal behavioral norms adaptable to diverse cultures, thereby reducing the intensity of human activities' demands on natural resources and energy. Indeed, the core value of AI lies not only in its ability to enhance the efficiency of energy, water, and land use but also in its capacity to promote environmental governance by improving the formulation and implementation effects of environmental protection policies through technological means.

Vinuesa et al. (2020) highlights that the rise of AI and its expanding influence across numerous fields leads to evaluate its role in achieving the Sustainable Development Goals (SDGs). Through a consensus-based expert consultation process, the paper finds that AI could facilitate the achievement of 134 SDG targets while potentially hindering the progress of 59 others. However, current research often overlooks certain key aspects. To ensure that the development of AI technology supports sustainable development, rapidly evolving AI requires accompanying regulatory insights and oversight mechanisms; otherwise, it might lead to deficiencies in transparency, safety, and ethical standards.

Rolnick et al. (2022) provides a comprehensive overview of how machine learning (ML) could aid in mitigating climate change. The study not only summarizes the current applications of ML in this field but also proposes many new ideas, from smart grids to disaster management. The authors calls for the establishment of a multidisciplinary collaboration platform to involve various stakeholders in addressing global warming.

Alzoubi and Mishra (2024) aims to explore the potentials and challenges of green AI initiatives. With the widespread application of AI technologies, especially generative AI, significant energy consumption issues have become a focal point, posing threats to sustainable development goals and environmental protection. The study extensively reviews existing literature, professional websites, and expert blogs, identifying and analyzing 55 green AI initiatives, which are categorized into six themes: cloud optimization, model efficiency, carbon footprinting, sustainability-focused AI development, open-source initiatives, and green AI research and community.

Rohde et al. (2024) points out that with the wide application of AI systems, the social, environmental, and economic impacts have become increasingly complex, necessitating a multidimensional perspective to understand and assess them. The article aims to provide a comprehensive evaluation framework for sustainable AI, namely a set of “Sustainability Criteria and Indicators for Artificial Intelligence Systems (SCAIS),” comprising 19 sustainability criteria and 67 related indicators to support the responsible development and application of AI technology.

Nishant et al. (2020) argues that artificial intelligence (AI) not only reshapes business operations and industrial landscapes, but also hold the potential to address critical societal issues, including sustainability. They point out that the core value of AI lies not only in its ability to enhance the efficiency of energy, water, and land use but also in its capacity to promote environmental governance by improving the formulation and implementation of environmental protection policies through technological means. Vinuesa et al. (2020) highlights that the rise of AI and its expanding influence across numerous fields could facilitate the achievement of several SDG targets while potentially hindering the progress others. Goralski and Tan (2020) underlines that AI presents both opportunities and risks. By means of three case studies, it analyzes AI's impact on management and leadership development, integrating perspectives from business strategy and public policy. Ahmad et al. (2021) explores the role of AI in optimizing the integration of energy sources into smart grids. It highlights that AI techniques surpass traditional models in handling big data, preventing cyberattacks, and enhancing energy efficiency and predictive maintenance. Rolnick et al. (2022) provides a comprehensive overview of how machine learning (ML) could help mitigating climate change. Moon (2023) points that while artificial intelligence (AI) has started to transform individual lives, business operations, and public services, there has been insufficient discussion regarding its role in contributing to social good. Alzoubi and Mishra (2024) explores the potentials and challenges of green AI initiatives. The study extensively reviews the existing literature, professional websites, and expert blogs, identifying and analyzing 55 green AI initiatives, which are categorized into six themes: cloud optimization, model efficiency, carbon footprinting, sustainability-focused AI development, open-source initiatives, and green AI research and community. Rohde et al. (2024) points out that with the wide application of AI systems, the social, environmental, and economic impacts have become increasingly complex, necessitating a multidimensional perspective to understand and assess them. Hernandez et al. (2024) investigates AI's role in achieving sustainable development goals, particularly in sustainable agriculture and waste management. Rane et al. (2024a)suggests that the transition to smart and sustainable industries through AI faces several challenges. Overcoming these challenges requires an integrated approach involving government policies, academic education, and industry innovation to foster a conducive environment for developing efficient, sustainable AI solutions. Rane et al. (2024b) suggests that integrating AI and ML into logistics and supply chain management is vital for boosting resilience and efficiency in the volatile global market. Rane et al. (2024c) highlights that the integration of artificial intelligence (AI), machine learning (ML), and deep learning (DL) is pivotal in the development of smart and sustainable cities and infrastructure, driving efficiency, sustainability, and livability. Habib and Mourad (2024) uses generalized least-squares (GLS) regression estimator and dynamic analysis techniques on a sample of 406 firms to examine the impact of ESG practices during the coronavirus crisis. Habib (2024) explores the connection between real earnings management (REM), ESG performance (ESGP), financial performance (FP), and total enterprise value (TEV) using multiple methods including PLS-SEM and regression analyses. Their findings reveal that REM strategies lead to lower ESGP and TEV, whereas ESG strategies enhance both TEV and FP. Ali (2024) employs a Bayesian Belief Network (BBN) model to explore tillage adaptation for flood management in soils with varying organic carbon contents during winter wheat production, thereby aligning with the UN SDGs 12 and 13. Li and Guo (2024) proposes a framework for co-creative communication to address the complexity and potential conflicts within the UN Sustainable Development Goals (SDGs).

The topic of Corporate Social Performance is also relatively recent, and not so many papers are available, especially with a rigorous quantitative approach. Among them, the following should be considered.

Dorfleitner et al. (2015) empirically examines various rating methodologies for Corporate Social Performance (CSP) using Environmental, Social, and Governance (ESG) scores from three rating agencies. The research reveals a significant divergence in ESG measurement approaches. Billio et al. (2021) delves into the ESG rating criteria utilized by leading rating agencies, highlighting the absence of common standards in defining ESG characteristics, attributes, and benchmarks for each component. Habib (2023) investigates the relationship between business strategies, ESG performance, and bankruptcy probability using partial least squares structural equation modeling (PLS-SEM). The results indicate that firms with stronger cost leadership strategies exhibit higher ESG performance and lower likelihood of financial distress. Roy (2023) underlines that as sustainable investment becomes a key driver of capital allocation, the significance of Environmental, Social, and Governance (ESG) measures continues to grow. It proposes an ESG performance-based credit rating model that employs the Fuzzy Best-Worst Method (BWM) for determining weights. The research identifies the financial pillar as the most significant, accounting for 43% of overall importance, followed by environmental (24%), social (19%), and governance (14%) pillars. Kumar Roy et al. (2023) shows that for Small and Medium-sized Enterprises (SMEs), which often lack structured financial data management, existing credit rating systems face numerous difficulties when dealing with sparse data. To bridge this gap, this study extends the application of expert systems by proposing a multi-criteria credit rating system.

Based on the previous literature, in this paper we will consider the following research hypotheses: i) Machine learning models can identify a positive relationship between ESG factors and credit ratings; ii) Machine learning models can be compared, and chosen, by means of a Sustainable, Accurate, Fair and Explainable (S.A.F.E.) metrics, threby further improving the sustainability of credit ratings.

## 4 Methodology

As already discussed, the aim of this paper is to build machine learning models capable to identify a relationship between ESG scores and credit ratings and, then, compare such models in terms of their Sustainability, Accuracy, Fairness and Explainability (S.A.F.E.)

To this aim, in the next subsection we briefly describe four alternative ensemble machine learning models, which seem well suited to the examined research context, being the ESG scores themselves the result of an aggregation of different indicators. Specifically, we will consider random forest, gradient boosting, stacking and voting models.

We will then proceed with the description of the S.A.F.E. metrics that will be used to compare the models, and of the related software package.

### 4.1 Ensemble models

Ensemble learning is a technique that enhances predictive performance by combining multiple base models. Noticeable examples of such models are random forest and gradient boosting methods, which have proved to be state of the art non linear models in credit scoring, and a powerful alternative to logistic regression models. Both models exploit the diversity between their components: in random forest models subsequent trees are built by means of training samples that differ not only in terms of data points but also in terms of the employed variables; in gradient boosting subsequent trees are generated on the residuals from the previous models. In both cases, the ensemble is built using components that belong to the same class of models: specifically, tree models.

The notion of ensemble models can be extended to the aggregation of models from different classes: for example, random forest models with logistic regression, or with gradient boosting. In this case, the diversity among models is greater.

In the context of credit rating prediction, general ensemble models of this type offer several advantages: first, by aggregating predictions from multiple models, they can mitigate the risk of any one model overfitting the training data, thus enhancing the model's generalization ability. Second, the aggregated predictions from multiple models are typically more stable and accurate than those from a single model, as they can capture different features and patterns in the data, leading to improved prediction accuracy. Third, ensemble models exhibit lower sensitivity to outliers and noise, making them perform better with complex and irregular data, which in turn strengthens the robustness of the model.

In this paper, we consider two types of general ensemble models: Stacked Ensemble Model (SEM) and Voting Ensemble Model (VEM).

Stacked Ensemble Model (SEM) enhances predictive modeling by integrating the outputs of multiple base models through a meta-model. The process begins with training several diverse base models, such as neural networks, random forests, and gradient boosting trees, each capturing different aspects of the data. These base models then generate predictions on both the training and test datasets, forming new feature matrices that serve as inputs for the next step. A meta-model, such as a linear regression model, is subsequently trained using these new feature matrices, aiming to learn how to best combine the predictions of the base models. The final predictions are made by applying the trained meta-model to the test set. This approach leverages the strengths of different base models, improving the overall stability and accuracy of the predictions by synthesizing their outputs through the meta-model.

Voting Ensemble Model (VEM) involves generating the final prediction result by either averaging the weighted predictions or selecting the majority vote from multiple base models. In this paper, we employ weighted averaging as, in regression tasks, the goal is to predict continuous values. In such scenarios, majority voting is not applicable because it is typically used for classification tasks, where the final prediction is determined by selecting the class that receives the most votes from the predictions of multiple models. Weighted averaging, on the other hand, can produce a continuous prediction value by summing up the weighted predictions from multiple models. The process involves first training multiple distinct base models and, then, using these models to make predictions on the test set. Finally, the predictions from multiple models are averaged with weights to generate the final prediction. One of the key advantages of this method is that it does not require additional training of a meta-model; instead, it directly processes the predictions from the base models. By weighting the predictions from multiple models, the instability of a single model can be reduced. The final prediction is a composite outcome of multiple models, making it easy to understand and interpret. Without loss of generality, in this paper we assign equal weights to the models.

### 4.2 The S.A.F.E. AI framework

Babaei et al. (2025) has recently proposed Sustainable, Accurate, Fair and Explainable (S.A.F.E.) metrics to evaluate the trustworthiness of AI output. The work extends a previous paper focused on AI applications in finance (Giudici and Raffinetti, 2023). Babaei et al. (2025) provides a model agnostic approach to assess machine learning, valid for all AI applications, independently on the underlying field domain, data and models, along with a Python software implementation: the safeaipackage, which allows full reproducibility.

The metrics proposed in their paper are consistent with each other, according to a common mathematical framework: the Lorenz curve. The Lorenz curve is a well known robust statistical tool, which has been employed, along with the related Gini index, to measure income and wealth inequalities. It thus appears as a natural methodology on which to build an integrated set of AI measurement metrics, allowing their integration in a unified decision theoretic framework.

Let us now briefly review the metrics. The requirement of accuracy refers to the measurement of the difference between the predicted and the actually observed (or expected) values of a response variable. Babaei et al. (2025) proposes to measure accuracy with the Rank Graduation Accuracy (RGA) which, in the binary case, is equal to the Area Under the Curve (AUC) but that, differently from the AUC, can be calculated for all types of variables. The RGA is a function of the area between the cumulative distribution of the observed values and the concordance curve: the same cumulative distribution, but ordered in terms of the predicted values.

The requirement of sustainability implies the the model outputs are stable under variations in the data and, in particular, when extreme data enter the available database. Babaei et al. (2025) propose to compare the concordance curves obtained using the ranks of the model and the ranks of a model in which the input data are perturbed. The area between the two concordance curves leads to a Rank Graduation Robustness measure (RGR) which can be interpreted as a measure of sustainability of a machine learning model.

The requirement of fairness implies that the predictions of AI applications do not present biases among different population groups, that typically derive from lack of representativeness and/or lack of quality of group specific data. To measure the fairness of AI applications, Babaei et al. (2025) proposes to compare the concordance curve of the predictions, with and without the protected variable which may lead to a bias This leads to a Rank Graduation Fairness (RGF) metric that will be, differently from the available metrics, model agnostic, thereby allowing comparison of fairness between different machine learning models.

An important advantage of the S.A.F.E. model just described is that all four proposed metrics are based on the same notion of variability, derived from the Lorenz curve. They can therefore be similarly normalized to [0,1] and integrated in a single [0,1] measure that can assess the trustworthiness of any AI application.

We now describe in more detail the mathematical comcepts behind the S.A.F.E. metrics. Let *Y* be a statistical variable to be predicted. In the machine leaning context, we observe *n* observations of *Y* in a test set, which will be employed to assess whether the predictions for the same observations, obtained from a machine learning model trained on a set of *m* observations, are Sustainable, Accurate, Fair and Explainable.

To this aim, the observed *Y* in the test set can be employed to build their Lorenz curve *L*, arranging the *Y* values in a non-decreasing sense. For *i* = 1, …, *n*, the Lorenz curve (Lorenz, 1905) can be defined by the pairs: $\left(i/n,\overset{i}{\underset{j=1}{\sum }}y_{r_{j}}/\left(nȳ\right)\right)$, where *r*_*j*_ indicates the non-decreasing ranks of *Y* and ȳ indicates the mean of *Y*.

The same *Y* values can also be used to build the dual Lorenz curve, $L_{Y}^{\prime }$, ordering the *Y* values in a non-increasing sense. For *i* = 1, …, *n*, the dual Lorenz curve can be defined by the pairs: $\left(i/n,\overset{i}{\underset{j=1}{\sum }}y_{r_{n+1-j}}/\left(nȳ\right)\right)$, where *r*_*n*+1−*j*_ indicates the non-increasing ranks of *Y*.

Now considering the predicted values for the *Y* values, which are obtained from a machine learning model. We can denote them by Y^*^. Let $r_{i}^{*}$, for *i* = 1, …, *n*, indicate the non-decreasing ranks of Y^*^.

To measure the divergence between *Y* and Y^*^, with a function which is based on the data and model independence, Giudici and Raffinetti (2024) proposes to calculate a concordance curve *C* = *C*(Y, Y^*^) ordering the *Y* values not in terms of their ranks, but with respect to $r_{i}^{*}$, the ranks of the Y^*^ values. More formally, for *i* = 1, …, *n*, a concordance curve is defined by the pairs: $\left(i/n,\overset{i}{\underset{j=1}{\sum }}y_{r_{j}^{*}}/\left(nȳ\right)\right)$, where $r_{i}^{*}$ indicates the non-decreasing ranks of Y^*^.

The introduction of the concordance curve has led (Giudici and Raffinetti, 2024) to introduce a Rank Graduation Accuracy measure (RGA), defined by the area between the $L_{Y}^{\prime }$ and the *C* curve, divided by the area of the Lorenz Zonoid. They also show that RGA is equivalent to the well-known Area Under the Curve (AUC), when *Y* is binary, with the advantage that RGA can be calculated in a similar way and also when *Y* is ordinal or continuous.

The other metrics, RGR, RGF and RGE can be defined in a similar manner, as detailed in Babaei et al. (2025).

## 5 Results

In this section we describe the employed data and the results from the application of the machine learning models and the S.A.F.E: metrics to them.

### 5.1 Data

We consider annual balance sheet data from a large sample of italian small and medium enterprises, covering the period from 2020 to 2022. The data source is the Modefinance database, a FinTech company accredited as a Credit Rating Agency by the European Securities and Markets Authority. The dataset includes ESG scores derived from indicators of environmental sustainability, social responsibility, and governance practices, along with financial metrics such as revenue, profit, assets, and liabilities, taken from the balance sheet of these companies.

Before analyzing the data, we have cleaned the data by removing rows that contain zero or null values, to improve data quality. Subsequently, the credit ratings have been transformed into numerical values, according to the conversion rule that we describe below, without loss of generality.

We need to ensure that the ratings from AAA to DDD are decreasing and that the difference between each rating is consistent. For example, we can assume that AAA corresponds to 95 and DDD to 5. Then, since there are a total of 13 ratings, the space between each rating can be assumed to be equal to $\frac{95-5}{13-1}=\frac{90}{12}=7.5$. The result of the numerical coding is represented in Table 1.

**Table 1:** Credit rating conversion.

**Credit rating**	**Numerical value**
AAA	95.0
AA	87.5
A	80.0
BBB	72.5
BB	65.0
B	57.5
CCC	50.0
CC	42.5
C	35.0
DDD	27.5
DD	20.0
D	12.5

We remark that the described numerical coding method is a simplified example; other codings may be specified, without altering the methdology we are going to describe. After this transformation, the response (dependent) variables are three variables, named MORE_evaluation_Score (the name assigned by the rating agency which issues the ratings), for the years 2020, 2021, and 2022.

The explanatory variables that can be employed to predict the credit ratings are described in Table 2. They include both ESG factors, assessed by the rating agency, and financial ratios, taken from the publicly available balance sheets of the companies. While the former are normalized and take values in [0,1], the latter are real numbers.

**Table 2:** Explanatory variables.

**ESG factors**	**Financial ratios**
ESG_score	Total_assets_EUR
final_normalized_score_env	Current_assets_EUR
final_normalized_score_social	Shareholders_funds_EUR
final_normalized_score_ governance	Current_liabilities_EUR
	Operating_revenue_Turnover_EUR
	Operating_profit_loss_EBIT_EUR
	Profit_loss_for_the_period_Net_income_EUR
	EBITDA_EUR

### 5.2 Exploratory analysis

**Figure 1:**
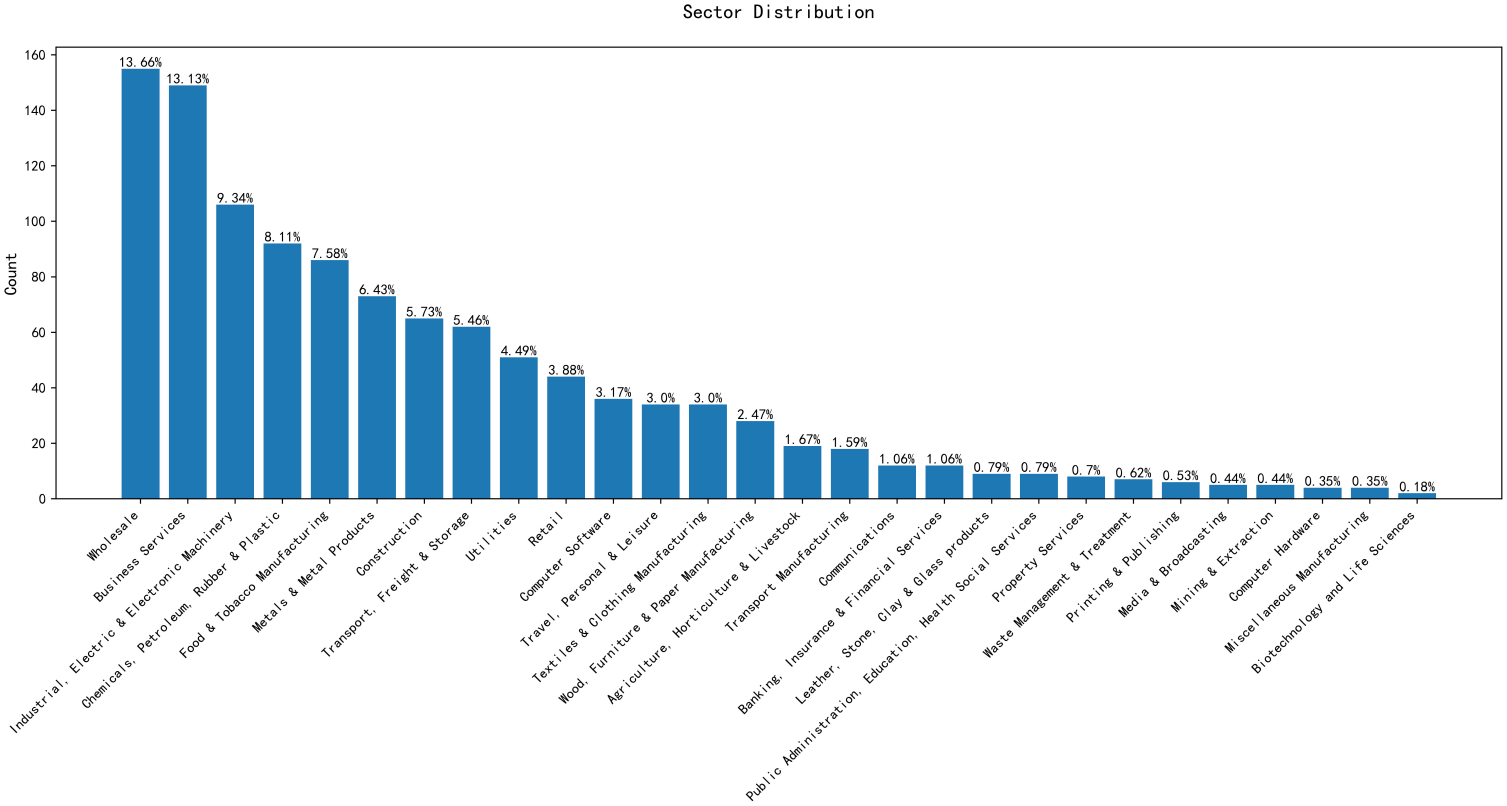
Distribution of companies by sectors.

Figure 1 represents the distribution of companies by sector. The Sector of belonging is another potential predictor variable.

From Figure 1, we can observe that certain sectors exhibit a higher number of companies. For instance, Business Services and Wholesale have the largest number of entities, reaching 149 and 155, respectively. In contrast, the Biotechnology and Life Sciences, Mining & Extraction, and Miscellaneous Manufacturing industries have few entities.

**Figure 2:**
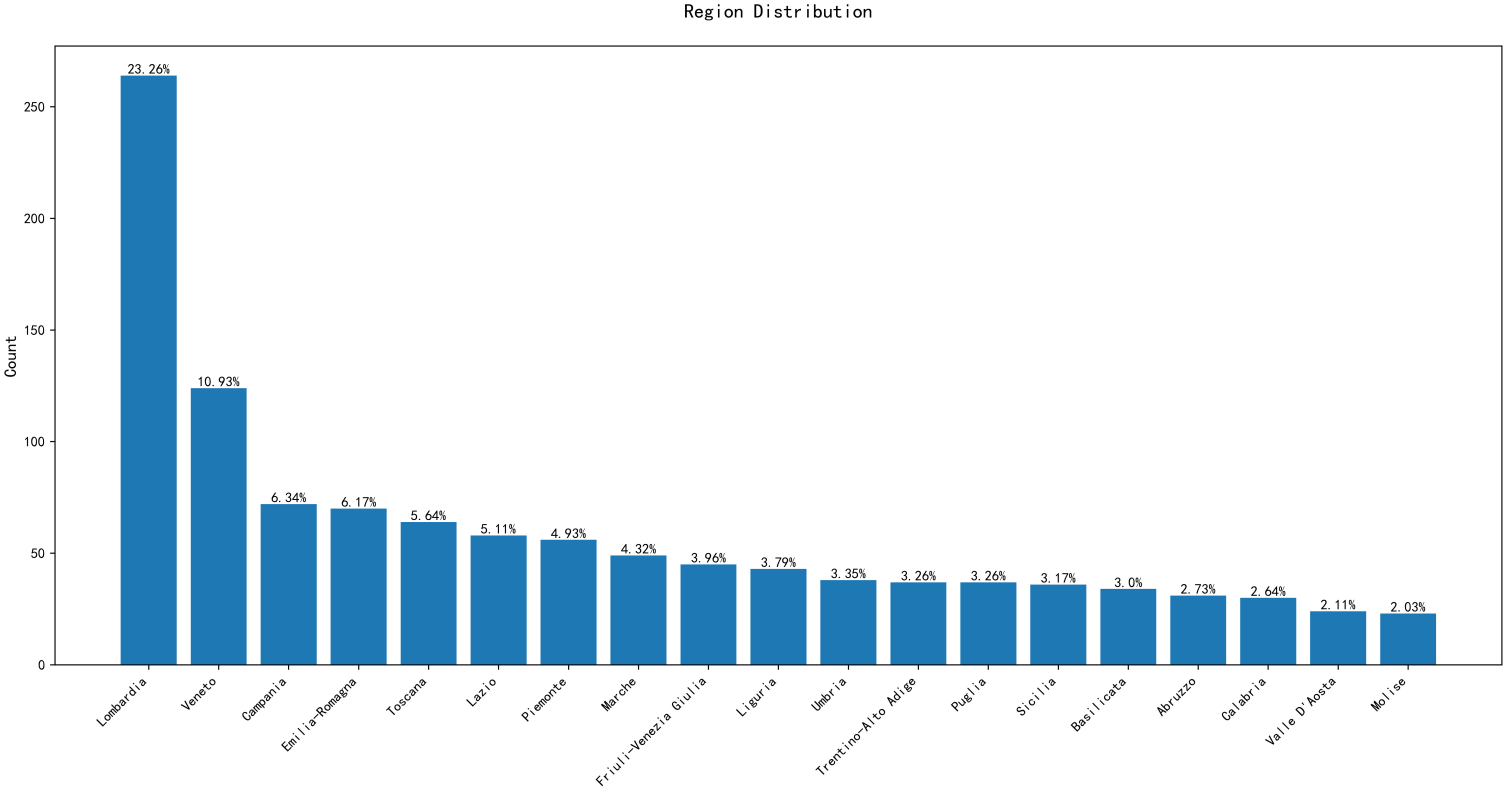
Distribution of companies by regions.

Figure 2 represents the distribution of companies along the twenty italian regions, another explanatory variable of interest.

Figure 2 shows that the majority of the companies in the dataset are concentrated in the Lombardia region, with a total of 264 entities; while the Molise region has the fewest, with only 23. Additionally, regions like Lazio and Veneto also show a significant number of data points. This geographical distribution reveals the varying levels of economic activity across different areas.

**Figure 3:**
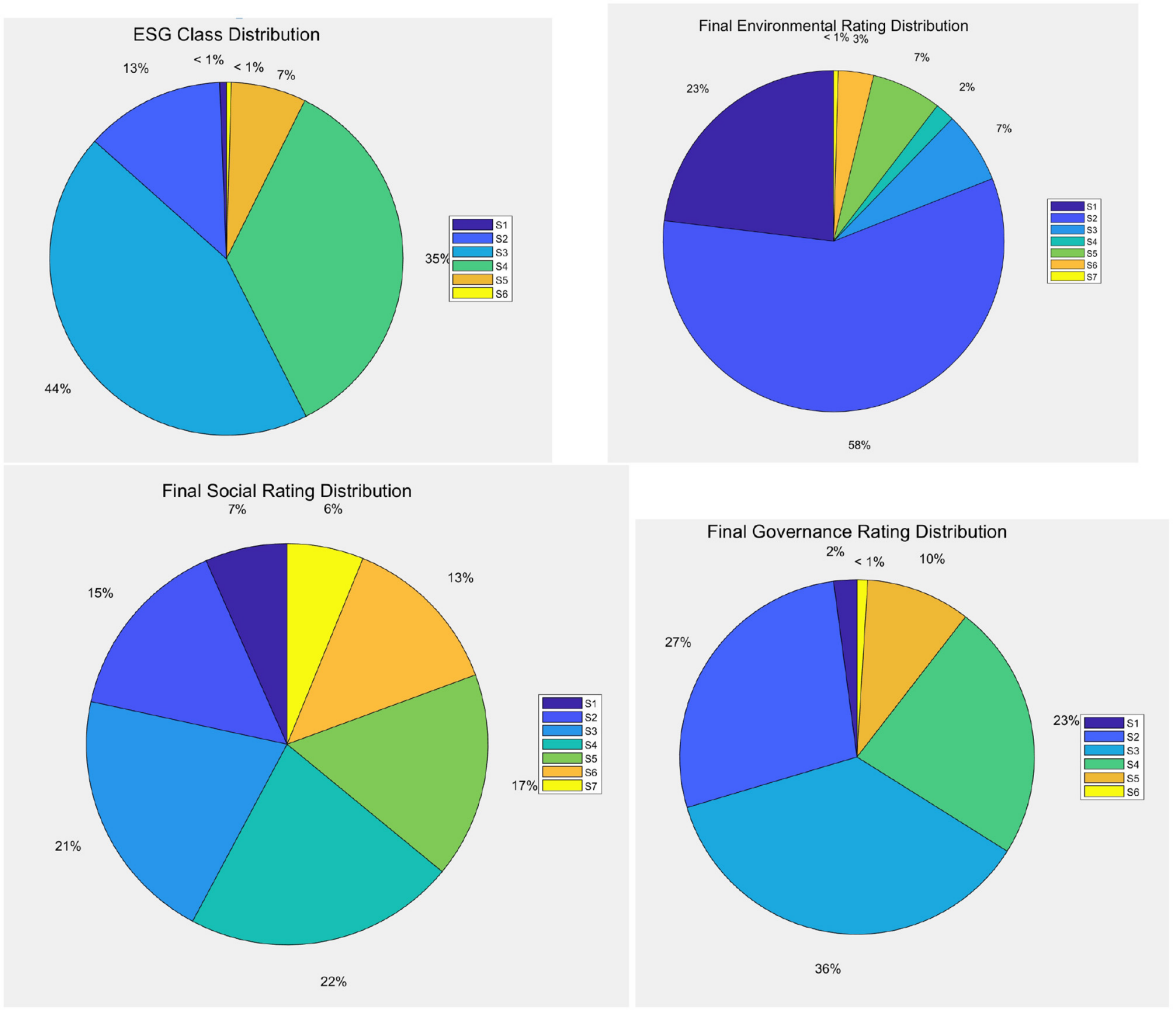
Distribution of companies by ESG classes, by environmental, social, and governance scores (clockwise).

Figure 3 represents the distribution of companies in terms of ESG scores, and by their components: Environmental scores, Social scores, Governance scores.

Figure 3 illustrates that, according to the ESG classification, the S3 and S4 categories include the majority of companies, 500 and 400, respectively, indicating that most companies are at a moderate level in terms of ESG performance. Meanwhile, the S1 (highest) and S6 (lowest) categories have relatively few entities, with only 7 and 5, respectively. This distribution suggests that although there are a few companies with exceptionally good or poor ESG performance, the majority still have considerable room for improvement. When discussing how ESG scores affect credit ratings, it would be beneficial to focus on those companies with better ESG performance (such as S1 and S2 categories) and analyze whether they enjoy higher market credibility and lower cost of capital. By doing so, the relationship between good ESG management and financial stability and long-term value creation can be more clearly demonstrated.

Figure 3 also reveals that, from the environmental, social, and governance perspectives, S2 is the most common rating for both environmental and social aspects, while in the governance rating, S2 and S3 take up a larger proportion. This indicates that most companies perform reasonably well in environmental protection and social responsibility, but may face more challenges in corporate governance. For investors, a robust corporate governance structure is often a key factor in investment decisions. Therefore, when exploring the impact of ESG scores on credit ratings, it is important to delve into the significance of each dimension, particularly the performance in the governance aspect, as effective governance structures are often seen as crucial for reducing business risks and enhancing transparency. Moreover, specific case studies could be used to illustrate how high ESG rated companies maintain their reputation through strengthened internal management and external communication, ultimately achieving a better credit status.

**Figure 4:**
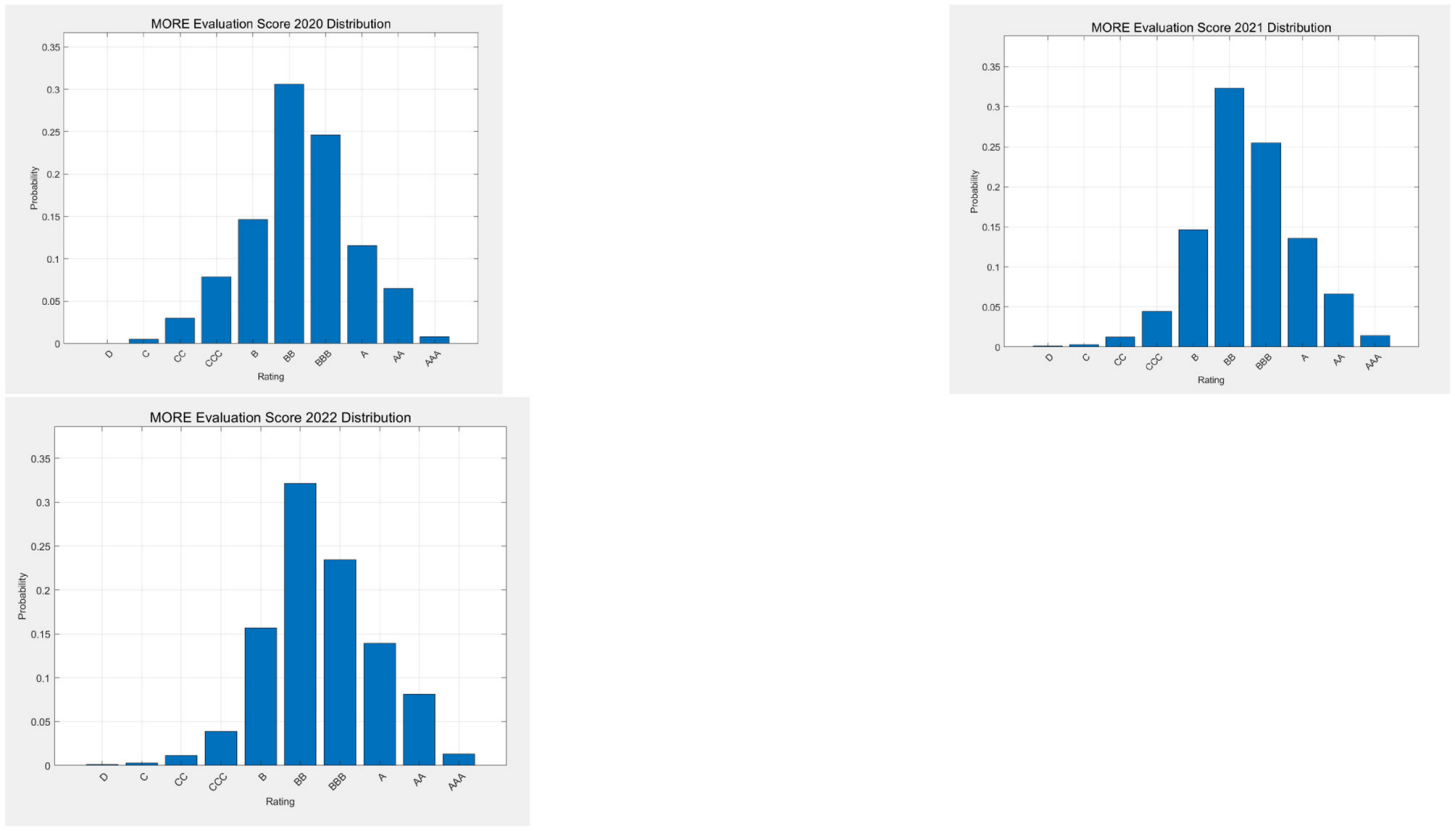
Distribution of companies by 2020, 2021, and 2022 credit ratings; from left to right.

Figure 4 displays the distribution of the companies in terms of credit ratings, for the considered years, as assigned by the MORE credit rating of the rating agency.

Figure 4 shows that the distribution of the credit ratings is quite stable over time, and is somewhat skewed to the right.

### 5.3 Empirical findings

Before applying the machine learning models, we have performed and extensive preprocessing, including extracting ESG scores and financial indicators from the raw dataset as explanatory variables, and credit ratings as the response variable. For categorical variables, one-hot encoding was applied to ensure that the model could correctly interpret the information. For numerical explanatory variables, standardization was performed to eliminate the effects of different scales among variables.

We are going to employ data from 2020 to forecast the credit ratings for 2021; and data from 2020 and 2021 to forecast the credit ratings for 2022. By combining 2020 and 2021 data, the experiments will explore whether integrating multiple time points enhances predictive accuracy for 2022 credit ratings. Additionally, the 2020 data will be used to forecast 2022 credit ratings, extending the prediction horizon to examine how well historical data can predict more distant credit ratings, two years into the future.

To objectively evaluate the models' predictive performance, we will use the Mean Square Errors and the Coefficient of Determination (R-squared) as evaluation metrics and, later in the paper, with the SAFE AI metrics introduced in the previous section.

As models to be compared, we have chosen the two state of the art non linear models for credit ratings: random forests and gradient boosting, as well as the two general ensemble models described in the previous Section.

#### 5.3.1 Random forest and gradient boosting tree

The results from the application of a random forest model and of a gradient boosting model are shown in Table 3.

**Table 3:** Prediction performance metrics for random forest and gradient boosting tree.

**Metric**	**Random forest**	**Gradient boosting trees**
MSE (using 2020 data to predict 2021)	64.54	61.4790
MSE (using 2020 data to predict 2022)	101.205	103.5172
MSE (using 2021 data to predict 2022)	64.9365	61.4940
MSE (using 2020 and 2021 data to predict 2022)	60.414	48.0326
R-squared (using 2020 data to predict 2021)	0.3942	0.42293
R-squared (using 2020 data to predict 2022)	0.081435	0.06045
R-squared (using 2021 data to predict 2022)	0.41062	0.44186
R-squared (using 2020 and 2021 data to predict 2022)	0.45167	0.56404

From Table 3, columns 2, note that, when using 2020 data to predict 2021 credit ratings, the model's MSE is 64.54 and R-squared is 0.3942, indicating that the model has some predictive capability, but limited explanatory power. When attempting to predict 2022 credit ratings using 2020 data, the model's MSE increases to 101.205 and R-squared decreases to 0.081435, suggesting that long-term predictions are more challenging, especially when using older data. However, when using 2021 data to predict 2022 credit ratings, the model's performance significantly improves, with a MSE of 64.9365 and an R-squared of 0.41062, demonstrating that using more recent data can effectively enhance prediction accuracy. Finally, combining 2020 and 2021 data to predict 2022 credit ratings further reduces the MSE to 60.414 and increases the R-squared to 0.45167, proving the effectiveness of integrating data from multiple time periods in improving prediction accuracy.

In summary, the application of the random forest model demonstrates that short-term predictions of credit ratings based on ESG factors (along with financial variables) are more accurate than long-term predictions, and that combining data from multiple time periods can significantly enhance the model's predictive capability. These findings deepen the understanding of how ESG scores influence credit ratings and provide valuable directions for future research on sustainability.

Table 3, columns 3 compares the prediction performance of the Gradient Boosting tree model under different time periods. When using 2020 data to predict 2021 credit ratings, the model's MSE is 61.4790 and R-squared is 0.42293, indicating that the model has a slightly better explanatory power compared to the random forest model. When attempting to predict 2022 credit ratings using 2020 data, the model's MSE increases to 103.5172 and R-squared decreases to 0.06045, suggesting that long-term predictions are again more challenging. However, when using 2021 data to predict 2022 credit ratings, the model's performance significantly improves, with an MSE of 61.4940 and R-squared of 0.44186, demonstrating that using more recent data can enhance prediction accuracy. Again, the gradient boosting trees model performs slightly better than the random forest model. Finally, combining 2020 and 2021 data to predict 2022 credit ratings further reduces the MSE to 48.0326 and increases the R-squared to 0.56404, proving the effectiveness of integrating data from multiple time periods in improving prediction accuracy. Also in this case, the gradient boosting trees model outperforms the random forest model significantly.

In summary, the gradient boosting method confirms that short-term predictions are more accurate than long-term predictions, and that combining data from multiple time periods can significantly enhance the model's predictive capability. Additionally, the gradient boosting trees algorithm shows a good performance, and outperforms the random forest model. The Table 4 summarizes the obtained results, in a comparative manner.

**Table 4:** Model performance metrics.

**Prediction scenario**	**MSE**	**R-squared**
Using 2020 data to predict 2021	GBT (61.4790) < RF (64.54)	GBT (0.42293) > RF (0.3942)
Using 2020 data to predict 2022	RF (101.205) < GBT (103.5172)	RF (0.081435) > GBT (0.06045)
Using 2021 data to predict 2022	GBT (61.4940) < RF (64.9365)	GBT (0.44186) > RF (0.41062)
Using 2020 and 2021 data to predict 2022	GBT (48.0326) < RF (60.414)	GBT (0.56404) > RF (0.45167)

Table 4 shows that the gradient boosting trees model outperforms the random forest model in most scenarios. Specifically, when using 2020 data to predict 2021 and 2022, and when leveraging both 2020 and 2021 data to predict 2022, the gradient boosting trees model demonstrates superior performance over the random forest model in terms of both MSE and R-squared (R-squared) metrics. Only in the case of predicting 2022 using only 2020 data the random forest model show a slight advantage. This suggests that, for the majority of predictive tasks, the gradient boosting trees model provides more accurate predictions.

We can conclude this part of the analysis stating that short term predictions using more recent data generally perform better, and that combining data from multiple time periods can significantly enhance the models's predictive capability. We can also conclude that the gradient boosting trees offers a better predictive accuracy with respect to a random forest model.

#### 5.3.2 Ensemble models

We now consider the ensemble models presented in the previous Section, the Stacked ensemble model and Voting ensemble model. The results of the predictions, using the same training and test samples as before, are reported in Table 5, column 2.

**Table 5:** Performance metrics for stacked ensemble model and voting ensemble model.

**Metric**	**Stacked ensemble model**	**Voting ensemble model**
MSE (2020 data to predict 2021)	26.0257	58.2021
MSE (2020 data to predict 2022)	39.2241	97.7470
MSE (2021 data to predict 2022)	27.2251	58.0859
MSE (2020 and 2021 data to predict 2022)	35.2000	48.4130
R-squared (2020 data to predict 2021)	0.75571	0.45369
R-squared (2020 data to predict 2022)	0.64399	0.11282
R-squared (2021 data to predict 2022)	0.75290	0.47280
R-squared (2020 and 2021 data to predict 2022)	0.68051	0.56059

Comparing Table 3 with Table 5 column 2 with note that, for all four predictions, the stacked ensemble model (SEM) shows a better performance, with substantially higher MSE and lower R-squared, compared to both Random Forest and Gradient boosting models. This makes it a very good choice for use in credit rating predictions.

In Table 5 column 3 we instead report the results from the Voting Ensemble Model. The results show that the Voting Ensemble Model (VEM) performs slightly better then the Random Forest or the Gradient Boosting model. However, it is clearly overperformed by the Stacked ensemble model, which shows the best performances.

### 5.4 SAFE AI metrics

We now present the application of the SAFE AI metrics, introduced in the previous Section, for a more comprehensive evaluation of the proposed models.

Specifically, we calculate the RGA (Rank Graduation Accuracy), RGR (Rank Graduation Robustness), RGE (Rank Graduation Explainability), and RGF (Rank Graduation Fairness). These metrics aim to comprehensively evaluate the overall sustainability of credit rating models, providing a scientific basis for their practical assessment, monitoring and mitigation.

For RGE, since the standardized final scores for the Environmental(E), Social (S), and Governance (G) aspects remain quite stable from 2020 to 2022, data processing for this part is relatively simplified. For RGF, we divide the samples into Northern and Southern regions, which are known to have different economic development, adding corresponding prefix identifiers (“N_” or “S_”). We then conduct a detailed analysis based on the geographical division and the industry categories of the different companies.

As machine learning models, we compare the Random Forest (RF), the Gradient Boosting Tree (GBT), the Stacked Ensemble Model (SEM), and the Voting Ensemble Model (VEM). For the Voting Ensemble Model, we use a simple average, where each model's weight is the same.

For the sake of clarity if, in the following, ESG scores are not included in the calculation of RGE, the latter is marked as RGE (ESG); if the influence of any single dimension–Environmental, Social, or Governance- is removed, they are named RGE (E), RGE (S), and RGE (G), respectively. Similarly, when the calculation of RGF ignores the industry factor, it is reported as RGF (Sectors); if the differences between the Northern or Southern regions are not considered, they are labeled as RGF (Region_N) and RGF (Region_S), respectively.

We present in Table 6 the resulting SAFE AI metrics of the models, using 2020 and 2021 data to predict 2022. Similar results can be shown for the other periods but we do not report them here, for lack of space.

**Table 6:** Model performance using data from 2020 and 2021 to predict 2022 credit ratings.

**RGB metric**	**RF**	**GBT**	**SEM**	**VEM**
RGA	0.50997	0.50814	0.51132	0.50950
RGR	0.52579	0.48267	0.50415	0.51030
RGE (ESG)	0.48961	0.49179	0.48884	0.49025
RGE (E)	0.49024	0.49449	0.49036	0.49188
RGE (S)	0.49060	0.49179	0.48903	0.49074
RGE (G)	0.49043	0.49200	0.48915	0.49078
RGF (Sectors)	0.49995	0.50000	0.49972	0.49998
RGF (Region_N)	0.49996	0.50000	0.49987	0.49999
RGF (Region_S)	0.49997	0.50000	0.49984	0.50000

We first consider the RGA, which extends the AUC for continuous responses, as that considered here, and measures predictive accuracy. From Table 6, the Stacked ensemble model (SEM) achieves an RGA value of 0.51132, outperforming all other tested models. It is followed by RF, with an RGA value of 0.50997. This indicates that when aiming to enhance prediction accuracy, RF and SEM serve as better options.

Then, we consider RGR, which measures a model's ability to maintain stability in the face of data variations. In this regard, RF demonstrates the strongest stability with an RGR value of 0.52579. Notably, while the VEM also performs well, its RGR is slightly lower compared to that of RF. The SEM shows a lower RGR value of 0.50415, indicating that it may be more susceptible to fluctuations in input data. Therefore, for applications which require high stability, it is more appropriate to prioritize the use of RF or VEM.

In evaluating the explainability of the models, we examine each model's performance, excluding different ESG factors to obtain different RGE values. Specifically, with the aim of assessing the impact of ESG scores on credit ratings, we consider RGE values when, respectively, the total ESG score, the environmental score, the social score, and the governance score are not included in the considered machine learning model.

For RF, the RGE values are 0.48961, 0.49024, 0.49060, and 0.49043, respectively. These results indicate that, even when certain key ESG indicators are removed, RF can still provide relatively stable predictive performance. However, compared to other models, its performance is slightly less impressive. GBT exhibits the highest RGE values in all four scenarios: 0.49179 without the total ESG score, 0.49449 without the environmental score, 0.49179 without the social score, and 0.49200 without the governance score. This not only indicates GBT's advantage in maintaining high explainability but also reflects its flexibility and robustness in handling complex datasets. SEM has RGE values of 0.48884, 0.49036, 0.48903, and 0.48915, respectively. Although SEM performs well in ensemble learning methods, its lower RGE values may suggest that the model's internal structure is more complex and difficult to interpret in terms of single variable influences. The VEM demonstrates high RGE values in every scenario evaluated: 0.49025, 0.49188, 0.49074, and 0.49078. This indicates that VEM maintains good predictive performance while also possessing a certain level of explainability. By calculating the average of RGE values for each model, our conclusion is that GBT takes the lead with an average RGE value of 0.4925175, followed by VEM (0.4909125), then RF (0.49022), and finally SEM (0.489345). Thus, in terms of RGE: Gradient Boosting Tree > Voting Ensemble Model > Random Forest > Stacked Ensemble Model. Therefore, if a powerful yet easily interpretable model is needed, A Gradient Boosting model is undoubtedly the best choice.

To assess the fairness of the models' output, we examine the impact of different geographical regions and industry backgrounds on model performance by means of the RGF values. Here, we focus on the RGF values when industry information and north/south regional data are not considered. GBT shows RGF values 0.50000 in all three scenarios, indicating that regardless of whether specific regional or industry information is included, GBT can produce consistent and fair results. However, compared to other models, its RGF values are slightly lower. RF has RGF values ranging from 0.49995 to 0.49997 which implies that RF remains a good fair model option in different. contexts. Similarly, the VEM also performs well, with RGF values consistently around 0.49999. This indicates that the VEM remains a highly fair model option in diverse contexts. The SEM has RGF values ranging from 0.49972 to 0.49987, showing some variability. Although it generally maintains good fairness, SEM is slightly less consistent across regions compared to GBT, RF and VEM. By calculating the mean of RGF values for each model, GBT leads with an average RGF value of 0.5000, followed by VEM (0.49999), RF (0.49996), and SEM (0.49981). Thus, in terms of RGF: Gradient Boosting Tree > Voting Ensemble Model > Random Forest > Stacked Ensemble Model. It is evident that Gradient Boosting models not only excel in explainability but they are also the optimal choice in terms of fairness.

Therefore, if higher prediction accuracy is the primary goal, then VEM is recommended. In scenarios where model stability is a priority, RF emerges as the preferred choice. If the main objective is to enhance model explainability and fairness, GBT is the best option. For applications requiring a balance between high explainability and fairness, while also considering other performance metrics such as MSE and R-squared, the VEM is also a worthy recommendation. Although RF and SEM perform well in these two aspects, it is slightly inferior to GBT and VEM, making it suitable for situations where explainability and fairness are important but not the highest priority. The SEM, which performs relatively weaker in these two key metrics, may not be the first choice for applications emphasizing model transparency and model bias.

### 5.5 Robustness

In this subsection we evaluate whether our findings are robust to alternative measures and estimations. In particular, we compare the obtained SAFE AI metrics with other metrics available in the literature which can provide a useful benchmark. Such metrics include the ROC (Receiver Operating Characteristic Curve) for assessing accuracy and SHAP (SHapley Additive exPlanations) values for model explainability. The comparison with standard metrics can enhance the robustness of our results, to assess the sustainability of AI applications.

In comparison with RGA the ROC curve reflects the model's ability to rank accurately the predicted values. When evaluating credit rating prediction models, the ROC is obtained by plotting the relationship between the True Positive Rate (TPR) and False Positive Rate (FPR) at various thresholds and calculating the area under the resulting curve. While is is known that RGA=AUC, it is of interest to calculate the coordinates of the ROC curve for specific threshod values, as this may give an indication about the possible prevalence of false positives over true positives.

For our data, Table 7 shows the results of the comparison.

**Table 7:** RGA and ROC at different thresholds.

**Metric**	**RF**	**GBT**	**SEM**	**VEM**
RGA	0.50997	0.50814	0.51132	0.50950
AUROC at threshold 70%	0.9385	0.8913	0.8124	0.8117
AUROC at threshold 85%	0.9774	0.9535	0.8869	0.8850

From Table 7 we can draw the following conclusions. RF exhibits the highest ROC values among all models, particularly reaching 0.9774 at the threshold of 85%, demonstrating its very good classification ability at high thresholds. GBT also shows good ROC values which are slightly lower than RF, and it can still maintain high levels at both thresholds. The SEM and VEM perform relatively lower in ROC, especially at the threshold of 70%, with values of 0.8124 and 0.8117, respectively, indicating their weaker ability to distinguish between positive and negative companies at low thresholds.

Thus, although the VEM performs best in terms of MSE and R-squared, its performance in ROC is inferior to that of RF and GBT. This suggests that while considering the overall accuracy of credit rating predictions, attention must also be paid to the model's classification ability at different thresholds. We therefore suggest that, when selecting the most suitable model, it is essential to consider a comprehensive range of performance metrics to ensure that the model delivers reliable credit rating predictions in practical applications.

In the context of explainability, the RGE metrics should be compared with Shapley values, the most used measure to obtain explainability of the machine learning output. RGE (Rank Graduation Explainability) measures the model's explainability by quantifying the impact on prediction rankings when specific features are removed. On the other hand, Shapley values, which originate from cooperative game theory, quantify the contribution of each feature to the predictions made by a machine learning model.

By comparing RGE with Shapley values, we can gain a comprehensive understanding of how the model's performance and explainability are affected by the inclusion or exclusion of different ESG factors, thereby better assessing the sustainability of a machine learning model.

In our results, we use Shapley (without ESG_score) to denote the Shapley values calculated without considering the ESG score, and similarly, we have Shapley (E), Shapley (S), and Shapley (G) for the cases where the environmental, social, and governance scores are excluded, respectively. The detailed results from the application of Shapley values are presented in Table 8.

**Table 8:** RGE and Shapley.

**RGE and Shapley**	**RF**	**GBT**	**SEM**	**VEM**
RGE (ESG)	0.48961	0.49179	0.48884	0.49025
RGE (E)	0.49024	0.49449	0.49036	0.49188
RGE (S)	0.49060	0.49179	0.48903	0.49074
RGE (G)	0.49043	0.49200	0.48915	0.49078
Shapley (ESG)	0.21417	0.22201	0.15801	0.15880
Shapley (E)	0.08127	0.64233	0.21299	0.21128
Shapley (S)	0.07772	0.11497	0.11503	0.11642
Shapley (G)	0.34705	1.92621	0.31991	0.31635

Table 8 shows that, in terms of the overall ESG score (ESG), GBT has the highest Shapley value at 0.22201, indicating the strongest overall dependency on the ESG score. The RF has the second highest Shapley value at 0.21417, suggesting a weaker dependency on the ESG score than that of GBT. The SEM and VEM have close Shapley values of 0.15801 and 0.15880, showing a relatively lower but similar dependency on the ESG score.

For the environmental score (E), GBT again has the highest Shapley value at 0.64233, indicating a higher emphasis on environmental factors. The SEM and VEM have close Shapley value of 0.21299 and 0.21128, showing a moderate emphasis on environmental factors. The RF has the lowest Shapley value at 0.08127, indicating a lower emphasis on environmental factors.

Regarding the social score (S), the VEM has the highest Shapley value at 0.11642, indicating a higher emphasis on social factors, followed by SEM (0.11503) and GBT (0.11497). These three values are close. The RF has the lowest Shapley value at 0.07772, indicating a lower emphasis on social factors.

For the governance score (G), the GBT has a significantly higher Shapley value at 1.92621, indicating an extremely high emphasis on governance factors. The RF model has the second-highest Shapley value with a score of 0.34705 and also indicating a high emphasis on governance factors. The SEM and VEM have close Shapley values of 0.31991 and 0.31635, showing a lower emphasis on governance factors.

It is important to note that Shapley values are used to explain the contribution of individual features to the model's predictions. Even when averaging Shapley values across multiple models, this operation still summarizes the feature impact on each model's predictions, rather than necessarily reflecting the final ensemble model's explanation. In the VEM, the predictions from different models are either weighted or simply averaged to produce the final prediction. In the SEM, we consider the weights of different models based on different R-squared. The influence of each model's prediction on the final result is based on its overall performance and accuracy, not just from individual feature contributions.

In summary, the results from the application of Shapley values confirm what obtained with the application of the RGE metrics. The detailed Shapley values analysis further helps in understanding the sensitivity of each model to ESG factor. For example, if the goal is to increase the model's focus on governance factors, choosing the GBT might be the better option. If a more balanced model is desired, the SEM or VEM could be considered. Furthermore, by delving into the Shapley values of different models, we can better explain the decision-making process of the models, thereby enhancing their transparency and interpretability.

## 6 Discussion

### 6.1 Theoretical implications

In this paper, we have proposed a methodology to improve the sustainability of Artificial Intelligence applications, embedding Environmental, Social, and Governance (ESG) factors into a set of alternative ensemble machine learning models.

Additionally, we have also shown how alternative models can be compared not only in terms of accuracy, but also in terms of explainability, fairness and robustness, thereby further improving the sustainability of artificial intelligence applications.

### 6.2 Managerial implications

We have exemplified our method for one of the most important applications of artificial intelligence in finance: the assessment of credit ratings for companies that ask for credit.

We have shown how different machine learning models can identify the relationship between ESG scores and credit ratings, capturing nonlinear effects. We have also shown that ensemble machine learning models are capable of providing valuable estimates.

The application of our methodology to a real use case shows that it is quite satisfactory and can thus be extended to further data and problems, in future research.

### 6.3 Limitations and future research agenda

The main limitation of our work is that the proposed S.A.F.E. metrics are applicable to artificial intelligence models for univariate responses that are numerical, ordinal or binary.

Future research should consider the extension to a multidimensional response and the consideration of non tabular data, as from the output of large language models.

## 7 Conclusions

We believe that model proposed in this paper is quite valid: from a theoretical viewpoint, as based on sound and consistent mathematical methods; from an empirical viewpoint, as the results in the previous Section have shown. We believe that both our research hypotheses have been validated: (i) it is possible to build machine learning models that measure the relationship between ESG scores and credit ratings; (ii) it is possible to make such models sustainable, accurate, fair and explainable (S.A.F.E.).

In the terminology of the existing AI recommendations and regulations, such as the American NIST AI Risk Management Framework and the European Artificial Intelligence Act, the model presented in this paper can be employed by to monitor, manage and mitigate the risks that derive from the applications of Artificial Intelligence, such as credit ratings.

The main strengths of our proposal are: (i) the modelisation of non linear effects of ESG factors on credit ratings, made possible by the employed machine learning models; (ii) the evaluation of the different models by means of a consistent set of metrics, all deriving from the same concept: the S.A.F.E. Ai metrics.

The main limitation of our proposal is that the metrics, to date, are designed for a unidimensional tabular response variable. An extension of the approach is necessary, in future research, to deal with more complex, multidimensional or non tabular responses.

## Data Availability

The original contributions presented in the study are included in the article/supplementary material, further inquiries can be directed to the corresponding author.
